# P-903. Sticker Reminder for IV-to-PO Fluoroquinolone Switching: Results from A Cluster-randomized Trial

**DOI:** 10.1093/ofid/ofaf695.1109

**Published:** 2026-01-11

**Authors:** Pinyo Rattanaumpawan, Jakaphan Sakchainan, Sumunthana Tunprayoon, Surangkana Samanloh

**Affiliations:** Faculty of Medicine Siriraj Hospital, Mahidol University, Bangkok, Krung Thep, Thailand; Faculty of Medicine Siriraj Hospital, Mahidol University, Bangkok, Krung Thep, Thailand; Faculty of Medicine Siriraj Hospital, Mahidol University, Bangkok, Krung Thep, Thailand; Faculty of Medicine Siriraj Hospital, Bangkok, Krung Thep, Thailand

## Abstract

**Background:**

One of easily-to-implement antimicrobial stewardship strategies is switching of intravenous antibiotic to oral antibiotic (IV-to-PO switching). Fluoroquinolone (FQ) is considered a good candidate for the IV-to-PO switching strategy because of its good oral bioavailability, safety, and tolerability.

**Methods:**

A cluster-randomized trial at 45 wards in Siriraj Hospital, Bangkok, evaluated a sticker reminder intervention to promote IV-to-PO fluoroquinolone (FQ) switching in hospitalized adults. Wards were randomized to standard care (the control group) or the sticker reminder intervention (the intervention group), where daily bedside evaluations triggered sticker placement on patient charts if switching criteria were met. Physician discretion determined the actual switch.

**Results:**

From July 1, 2020 – December 31, 2024, we enrolled 83 patients into the intervention group and 85 patients into the control group. The most common site of infection was lower respiratory tract infection, followed by urinary tract infection and soft tissue infection. Similar proportions of patients in the intervention group and the control group (81.93% vs 83.53%; p-value=0.78) met the IV-to-PO switching criteria. The proportion of IV-to-PO switching among those who met all IV-to-PO switching criteria of the intervention group was significantly higher than those in the control group (42.17% vs. 58.82%; p-value=0.03). There was no statistical difference in clinical cure rate, hospital mortality, length of hospital stays and duration of antimicrobial therapy.

**Conclusion:**

The sticker reminder intervention showed benefit on promoting the IV-to-PO switching. Based on these findings, additional qualitative studies to explore hesitation to use IV-to-PO switching among the prescribers is necessary.

**Disclosures:**

All Authors: No reported disclosures

